# A Rare Case of Intravascular Papillary Endothelial Hyperplasia of the Hypopharynx: A Case Report and Literature Review

**DOI:** 10.7759/cureus.88935

**Published:** 2025-07-28

**Authors:** Erika S Johnson, Jauntea M Maxey, Robert Brody, Paul Zhang, Nikolina Dioufa

**Affiliations:** 1 Pathology, Hospital of the University of Pennsylvania, Philadelphia, USA; 2 Otorhinolaryngology-Head and Neck Surgery, Hospital of the University of Pennsylvania, Philadelphia, USA; 3 Otorhinolaryngology-Head and Neck Surgery, Veteran's Administration Medical Center, Philadelphia, USA

**Keywords:** head and neck neoplasms, hypopharynx, intravascular papillary endothelial hyperplasia (masson's tumor), papillary lesions, vascular proliferation

## Abstract

Intravascular papillary endothelial hyperplasia (IPEH), also known as Masson’s hemangioma, is a rare benign vascular lesion characterized by an unusual pattern of endothelial proliferation within a thrombosed vessel. Histologically, IPEH is defined by prominent intraluminal papillary structures composed of a single layer of enlarged endothelial cells, lacking cytologic atypia, mitotic activity, and necrosis. Here, we report a rare case of IPEH identified in the hypopharynx of a 56-year-old male patient, incidentally, on imaging for evaluation of unrelated upper back pain. We describe the clinical presentation, histopathologic findings, differential diagnosis, and review of the current literature to enhance recognition and management of this entity in rare/unusual sites.

## Introduction

Intravascular papillary endothelial hyperplasia (IPEH), first discovered by Pierre Masson in 1923, is a rare, benign vascular lesion characterized by reactive proliferation of endothelial cells associated with an organizing papillary thrombus. Initially termed hemangioendotheliome végétant intravasculaire, it was later renamed by Clearkin and Enzinger in 1976 to its current designation. IPEH arises in various anatomical locations, including the skin, subcutaneous tissue, and mucous membranes, and most commonly affects the head and neck region, particularly the face and scalp [1-3], but has also been reported in less typical sites such as the buccal mucosa, tongue, masseter muscle, parotid gland, and orbit [4,5]. Involvement of deeper structures like the larynx, hypopharynx, and even intracranial sites remains exceptionally rare [6-10]. Based on our review, our case is the third reported case of IPEH arising in the hypopharynx worldwide [8].

Clinically, IPEH typically presents as a firm, sometimes tender mass, often associated with reddish-blue discoloration of the overlying skin or mucosa [2]. However, its nonspecific presentation frequently leads to misdiagnosis, with differential considerations including mucocele, hemangioma, pyogenic granuloma, and malignant entities such as angiosarcoma and Kaposi sarcoma [11-13]. Therefore, an accurate diagnosis hinges on careful histopathologic evaluation to prevent unnecessary aggressive treatment, which reveals characteristic intraluminal papillary structures lined by a single layer of plump endothelial cells without cytologic atypia, mitotic figures, or necrosis.

## Case presentation

**Figure 1:**
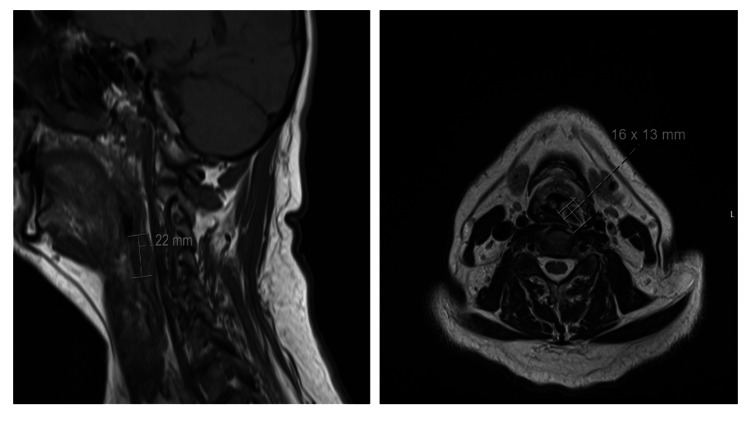
MRI of the C-spine revealed a 1.6 x 1.3 x 2.2 cm submucosal mass in the left posterior lateral hypopharynx (lateral and cross-section images).

Our patient is a 56-year-old male patient with a smoking history who presented with persistent neck pain, and a workup with an MRI of the C-spine revealed a 1.6 x 1.3 x 2.2 cm submucosal mass in the left posterior lateral hypopharynx, with a mild mass effect on the airway, suspicious for neoplasm (Figure 1). The mass was heterogeneous and enhancing, without evidence of internal vascularity, without extension to the vertebrae. There was also no pathologic lymphadenopathy noted.

The differential diagnoses of the mass, given the nonspecific characteristics, included squamous cell carcinoma, schwannoma, rhabdomyoma, and granular cell tumor. A PET/CT DOTATATE scan showed no somatostatin receptor neoplasm, and a CT chest revealed atelectasis and nodules without evidence of thoracic disease or malignancy. At the office, a direct laryngoscopy was performed. The oropharynx had no lesions concerning for malignancy. When the Dedo laryngoscope was inserted into the post-cricoid region and the hypopharynx was inspected, fullness in the submucosal region at the level of the thyrohyoid membrane was noted. The larynx was examined, revealing normal mucosa. Aspiration was attempted with a 23-G angiocath, and the lesion appeared solid without a fluid component. An incision of the mucosa revealed a vascular lesion in the submucosal space. A decision was made to surgically resect the tumor via a transoral robotic approach with limited pharyngectomy. Intraoperatively, the tumor was successfully and completely excised and submitted for histopathologic examination.

**Figure 2:**
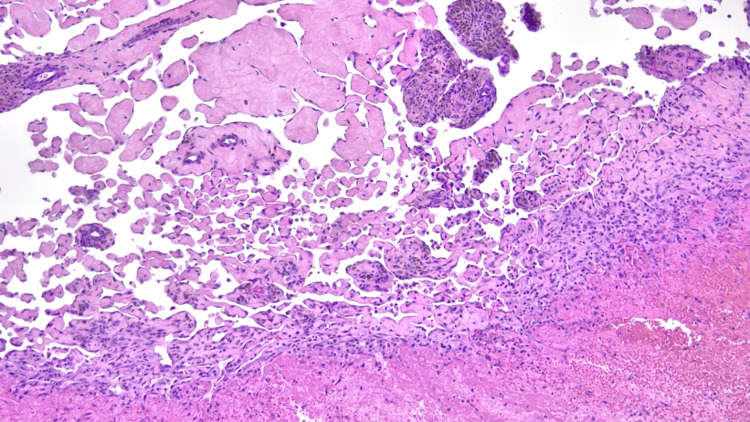
High magnification images show that the papillae consist of cores of fibrin or fibrous connective tissue and are lined by a single layer of plump endothelial cells. Thrombosis is seen at the bottom right corner of the image and numerous hemosiderin-laden macrophages, compatible with prior hemorrhage.

**Figure 3:**
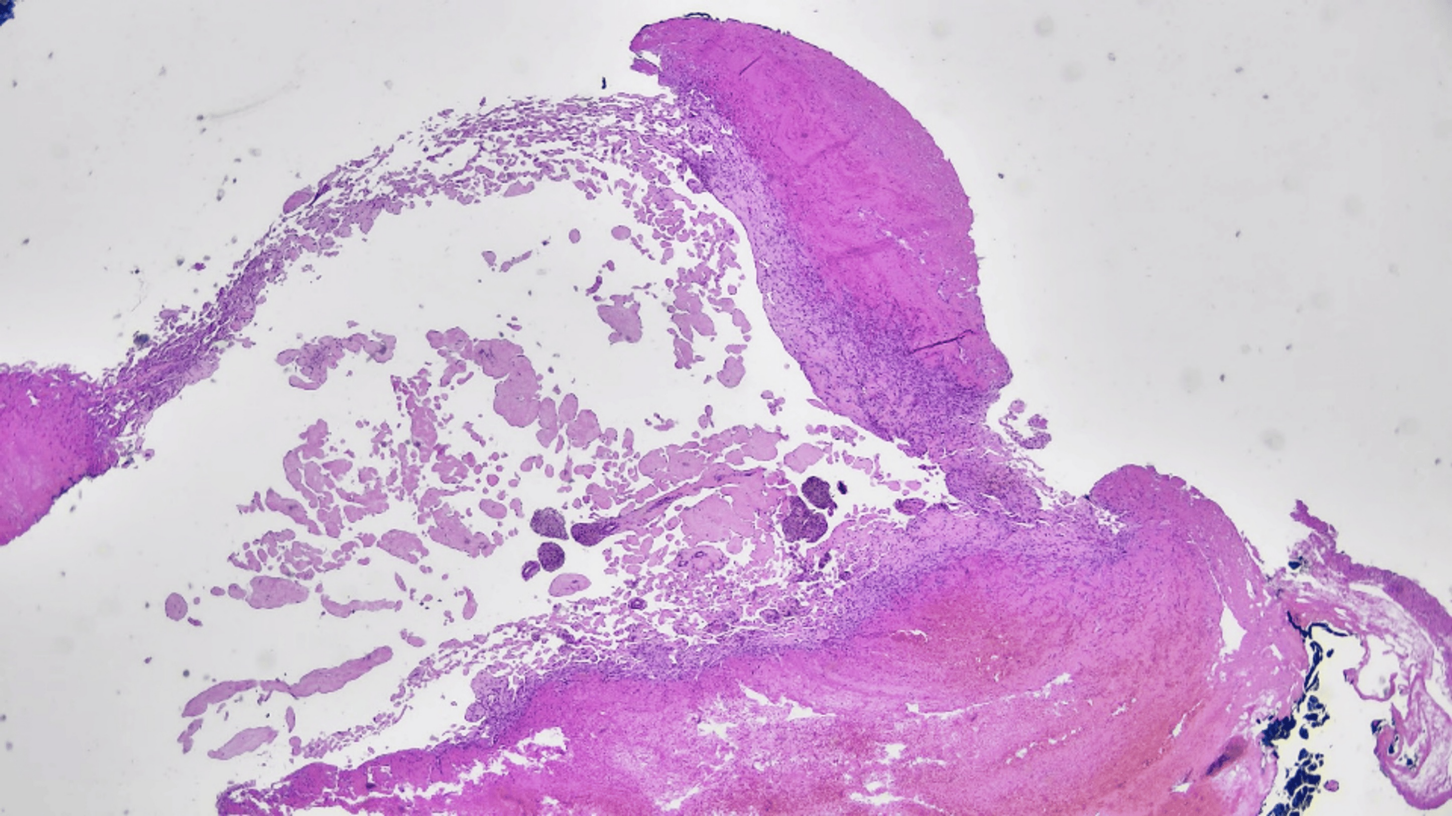
Low-power magnification of the lesion shows numerous papillary structures within a vascular space with surrounding hemorrhage and thrombosis.

Grossly, the tissue showed a reddish, rubbery mass, focally congested. Histological examination revealed dilated vascular spaces with numerous fibrovascular papillae lined by a single layer of plump endothelial cells, in association with organizing thrombus. There was no evidence of atypia or mitotic activity, confirming the diagnosis of IPEH (Figure 2 and Figure 3). Routinely, as in our case, Masson hemangioma is a histomorphologic diagnosis. Immunohistochemical stains, such as CD31, CD34, and ERG, can be used to highlight the endothelial cells lining the avascular or hyalinized papillae.

On his follow-up appointment, the patient reported recovering well, without any post-surgical complications. His unrelated cervical pain persisted, for which physical therapy was recommended.

## Discussion

IPEH, or Masson’s tumor, is a benign vascular lesion characterized by a reactive proliferation of endothelial cells forming papillary structures within the lumen of blood vessels. It is considered a variant of an organizing thrombus rather than a true neoplasm. Although the exact pathogenesis remains uncertain, factors such as prior trauma and hormonal influences have been suggested as contributing elements [1, 10]. Most of the reported cases are small in size (<5 cm), slow-growing lesions or swellings, superficially located, commonly in the subcutis in the head and neck area, and the upper extremities. Often, they can be incidental findings within thrombosed dilated blood vessels or vascular tumors examined for other underlying pathology.

IPEH has been classified into three types by Hashimoto et al.: (i) a pure/primary form that arises in dilated vessels, being the most common type; (ii) a mixed/secondary form that occurs in preexisting vascular abnormalities, such as varices, hemangiomas, lymphangiomas, pyogenic granulomas, and arteriovenous malformations; and (iii) the undetermined type, a rare extravascular hemangioma developing in the bed of an organizing hematoma [1]. Our case has features of the pure/primary form, arising within a dilated blood vessel and in association with an organizing thrombus.

Histologically, IPEH is characterized by the presence of numerous fibrovascular papillae lined by a single layer of plump endothelial cells, often associated with thrombi in varying stages of organization. Importantly, features of malignancy such as nuclear atypia, increased mitotic activity, and necrosis are absent. These histological characteristics are essential for differentiating IPEH from malignant vascular tumors like angiosarcoma, where infiltrative growth, cytologic pleomorphism, high mitotic rate, and necrosis are prominent [12-16]. 

Clinically and radiographically, IPEH lacks specific distinguishing features, particularly when arising in rare sites like the pharynx or larynx. As a result, it may mimic a wide spectrum of benign and malignant lesions, some vascular, prompting an aggressive surgical management under the suspicion of malignancy. Specifically with the case presented, given the clinical and radiographic concern for malignancy, a more extensive resection, namely a total laryngectomy and partial pharyngectomy, was the alternative recommended approach. This morbid procedure was avoided by performing a more limited transoral resection that allowed for complete excision of the lesion.

Furthermore, in our review of the literature, the mass-forming lesions, depending on the location, were read by radiology as lesions suspicious for carcinoma, sarcoma, or vascular neoplasm, both benign and malignant, prompting surgical resection for definitive diagnosis. Therefore, thorough histopathologic evaluation remains the cornerstone of accurate diagnosis. Table 1 summarizes the histopathologic features of the various entities (benign and malignant) that are included in the differential diagnosis of IPEH. Mucoceles can often be seen in the head and neck area, and mucicarmine special stain can easily confirm the presence of extravasated mucin in association with granulation tissue. However, in the differential diagnosis, many lesions are vascular in origin and will show some overlap with IPEH both on morphology and on immunohistochemistry. The endothelial markers CD31, CD34, and ERG will be positive in pyogenic granuloma, hemangioma, vascular malformation, Kaposi sarcoma, and angiosarcoma. On histology, angiosarcoma will have a more complex architecture, with infiltrating and anastomosing vascular channels lined by spindled to epithelioid endothelial cells with variable cytologic atypia. The endothelial cells may also have a multilayered appearance or appear free-floating within the vessel lumen. Immunohistochemistry for FLI-1 will be positive, and MYC can be positive in cases of radiation- or lymphedema-induced angiosarcomas [13]. For Kaposi sarcoma, the clinical history and presence of extravasated red blood cells are helpful, and diagnosis can be confirmed via human herpesvirus 8 (HHV8) immunostain [12].

**Table 1:** Summary of the clinical and histopathologic findings of the various entities included in the differential diagnosis of IPEH IPEH: intravascular papillary endothelial hyperplasia; HHV8: human herpesvirus 8; VVG: Verhoeff-Van Gieson

Entity	Clinical features	Histopathologic features	Key differences from IPEH	Immunohistochemical stains
Pyogenic granuloma (lobular capillary hemangioma) [2,3, 5,6]	Pedunculated, hemorrhagic, common on the gingiva, lips, and buccal mucosa	Lobular capillary proliferation, no vessel wall	Lack of intravascular organization, absence of papillary structures within a vessel	Positive for CD31, CD34, ERG, SMA and negative for HHV8 and GLUT-1
Kaposi sarcoma [2,3,12]	Red to bluish plaques/nodules, common on the palate, tongue, often multifocal, associated with HIV	Slit-like vascular spaces, spindle cells, hemosiderin deposition, infiltrative or nodular growth	Presence of spindle cells, slit-like vascular spaces, plasma cell infiltration, extravasated RBCs, numerous mitoses	Positive for CD31, CD34, ERG, HHV8; Negative for SMA, Desmin
Angiosarcoma [2,13,15, 16]	Rapidly enlarging mass, often cutaneous. Association with prior radiation and longstanding lymphedema	Infiltrative growth, nuclear atypia, frequent mitoses, necrosis	Cytologic pleomorphism and aggressive infiltration are absent in IPEH. Often, necrosis, increased mitoses, and solid growth are present in angiosarcoma.	Positive for CD31, CD34, ERG, FLI1, VEGF, Factor VIII, PanCK (focal), EMA (focal); Negative for HHV8
Hemangioma [2]	A bluish submucosal mass, common in infants	Well-formed vascular channels without endothelial proliferation, lined by a cytologically bland single layer of endothelial cells	Endothelial proliferation is only seen with secondary trauma; various types exist, including infantile, intramuscular, spindle cell, cavernous, anastomosing, and angiomatosis	Positive for CD31, CD34, ERG, GLUT-1 (infantile), FLI1; Negative for HHV8, SMA and TFE3
Mucocele [2]	Bluish, cyst-like swelling, common on the lower lip	Mucous-filled cavity lined by granulation tissue	Absence of endothelial proliferation and thrombus	Mucicarmine special stain
Endovascular papillary angioendothelioma [2,3,14]	Soft tissue mass, rare. Considered a borderline entity between hemangioma and angiosarcoma, with a favorable prognosis	Numerous interconnecting vascular channels with papillary projections or tuft-like structures. Endothelial cells with a classic hobnail or matchstick appearance.	Characteristic appearance of endothelial cells with apically placed nucleus. High nuclear to cytoplasmic ratio and moderate mitotic activity.	Positive for CD31, CD34, ERG, VEGFR, D2-40
Arteriovenous malformation [17]	Soft tissue mass, warm, red, pulsatile	Large tortuous, thick-walled arteries and veins, along with a small vessel component with a capillary-like appearance. Arteries may show focal loss of internal elastic lamina, while veins may show thickened walls.	Lack of intravascular organization, absence of papillary structures within a vessel	Positive for CD31, ERG, SMA (highlights muscularized vascular wall), and VVG stain (internal elastic lamina of arterioles)

Given the rare occurrence of IPEH in the head and neck area and hypopharynx [6-10], cases such as the one presented here emphasize the importance of recognizing this entity to avoid unnecessary extensive surgical interventions and adjuvant therapies. Complete surgical excision remains the treatment of choice, with an excellent prognosis and low recurrence risk, mainly associated with incomplete removal. 

To date, reports of hypopharyngeal IPEH remain exceedingly rare. Increased reporting of such cases is critical to expanding the clinical and histopathological spectrum of IPEH, particularly in unusual sites. Greater awareness among clinicians and pathologists will aid in ensuring accurate diagnosis and appropriate management, ultimately reducing the risk of overtreatment for a benign condition. 

## Conclusions

IPEH is a rare, benign vascular lesion that can closely mimic malignant neoplasms both clinically and radiologically, particularly when arising in uncommon locations such as the hypopharynx. Accurate diagnosis relies on careful histopathologic evaluation to differentiate it from more aggressive vascular tumors like angiosarcoma. Complete surgical resection is curative, with a low risk of recurrence when fully removed. Increased recognition and reporting of IPEH in rare anatomical sites are essential to broaden clinical awareness, refine diagnostic criteria, and prevent unnecessary aggressive treatment for this benign entity.
